# Supplementary material for: Molecular and Genomic Characterization of the Pseudomonas syringae Phylogroup 4: An Emerging Pathogen of Arabidopsis thaliana and Nicotiana benthamiana
Source: Microorganisms. 2022 Mar 24;10(4):707. doi: 10.3390/microorganisms10040707 (PMC9030749; doi:10.3390/microorganisms10040707)
Supplement: Supplementary file 1 [file microorganisms-10-00707-s001.zip › microorganisms-1639357-supplementary-Figures.pdf]

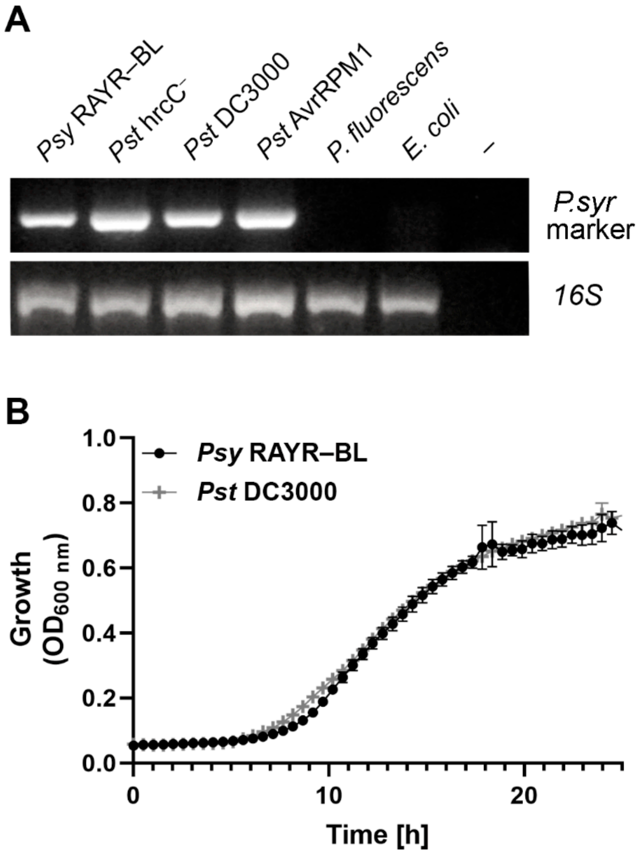

C

	Pst DC3000	*Psy* RAYR-BL	p-value
Carrying capacity	0.71±0.03	0.72±0.03	0.595
Growth rate (μ, h-1)	0.38±0.04	0.34±0.04	0.314
Doubling time (h)	2.03±0.23	1.70±0.09	0.078

Figure S1: *P. syringae Psy* RAYR-BL characterization. (A) Identification of *P. syringae* marker gene. Genomic DNA was extracted from *Psy* RAYR-BL, *Pst* DC3000, *Pst* AvrRPM1, *P. fluorescens* and *E. coli*. PCR was performed with oligonucleotides that allow the amplification of a Pst marker gene [50]. To evaluate the DNA extraction, the *16S* gene was amplified. Oligonucleotides are listed in **Table S12**. **(B)** The kinetics growth dynamics of *Pst* DC3000 and *Psy* RAYR-BL are similar on King's B media. We evaluate the growth of the *Pst* RAYR-BL strain compared to the *Pst* DC3000 strain. A 24 well plates were inoculated with the bacteria on King's B media and the bacterial growth was measured. The data are expressed as the mean of Absorbance ($OD_{\lambda 600}$) of three replicates ± SD. **(C)** The Carrying capacity, Growth rate (μ), and Doubling time (h) were calculated for each strain. There were no differences between the strains according a t-test. The p-value is indicated on the last column.

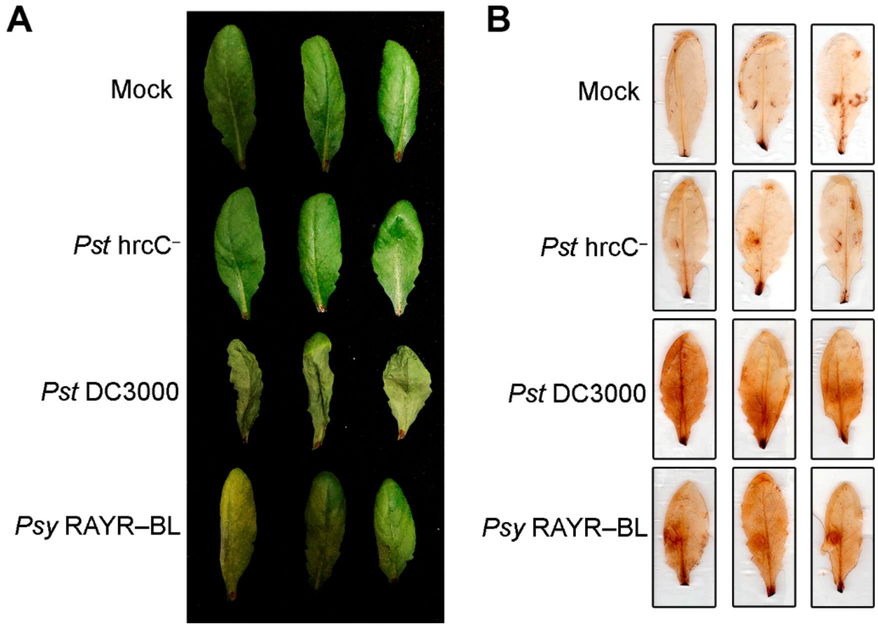

Figure S2: *Arabidopsis thaliana* ecotype Col-0 plants are susceptible to the infection with the *Pseudomonas syringae* RAYR-BL strain. 4-weeks old plants were syringe-inoculated with *Pseudomonas syringae* RAYR-BL (*Psy* RAYR-BL), *Pseudomonas syringae* pv *tomato* DC3000 (*Pst* DC3000), *Pseudomonas syringae* hrrC$^-$(Pst hrcC–or $MgCl_2$ 10mM as control (Mock). (A) The disease phenotype was visualized after 48 h post-inoculation. **(B)** H_2O_2 levels were detected using DAB staining on the inoculated leaves 48 hour post-inoculation.

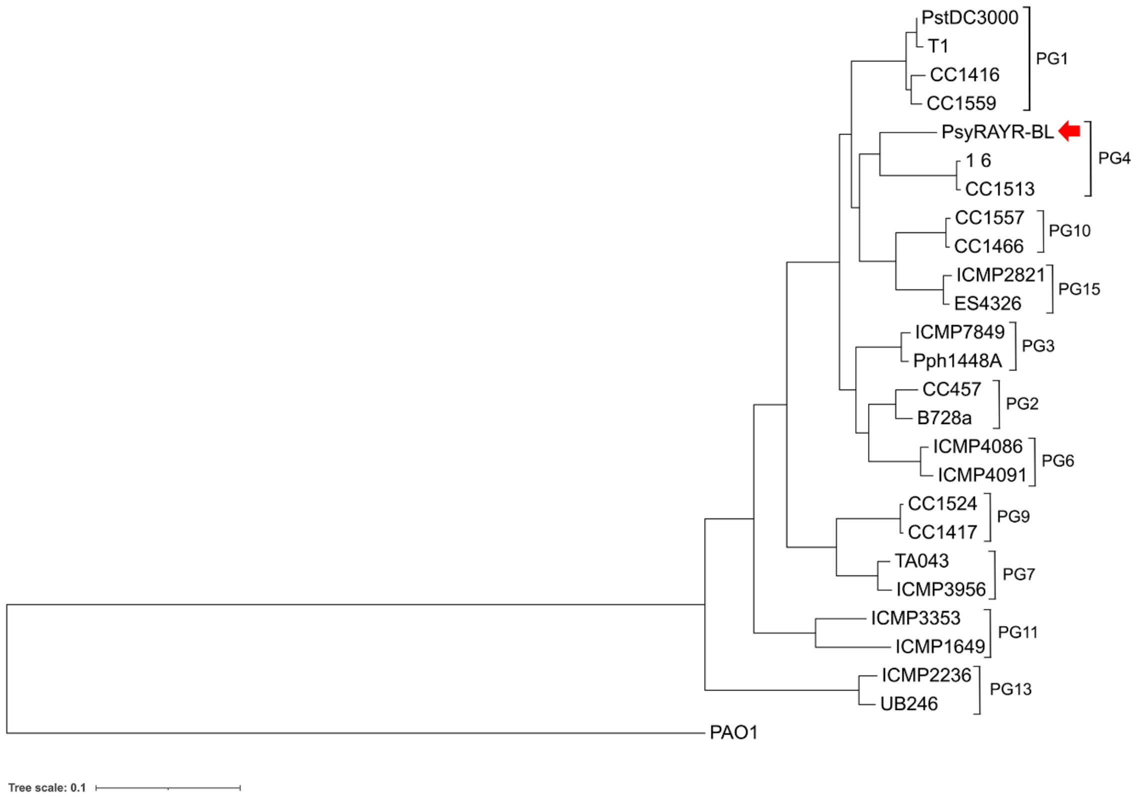

Figure S3: Phylogenetic relationships of *Psy* RAYR-BL with other *P. syringae* strains based on 9 housekeeping genes. Phylogenetic Maximum Likelihood tree was estimated based on the concatenated partial nucleotide sequence of 9 housekeeping genes: *aroE* (encoding shikimate dehydrogenase), *gapA* (glyceraldehyde-3-phosphate dehydrogenase A), *gltA* (citrate synthase), *gyrB* (DNA gyrase subunit B), *pgi* (glucose-6-phosphate isomerase), *pyrC* (dihydroorotase), *recA* (protein RecA), *rpoB* (DNA-directed RNA polymerase subunit beta), *rpoD* (RNA polymerase sigma factor RpoD). In total 13,636 positions where considered. The phylogenetic tree was constructed with 24 strains of the of the *Pseudomonas syringae* complex (representing 11 phylogroups) plus *Psy* RAYR-BL. *Pseudomonas aeruginosa* PAO1 was used as an outgroup.

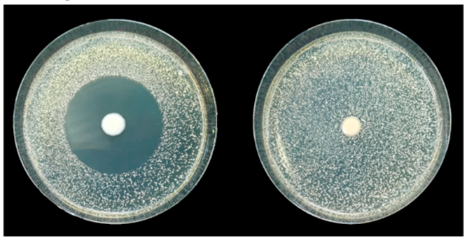

Figure S4: Evaluation of syringomycin/syringopeptin production on *Psy* RAYR-BL and *Pst* DC3000. A drop of saturated of *Pst* DC3000 and *Psy* RAYR-BL bacterial culture was placed in the center of a King's B semisolid plate. The plates were incubated during 24 hours at 28°C. Then, a suspension of *S. cerevisiae* (that is susceptible to the syringomycin/syringopeptin toxins) were sprayed on the plates. After 24 hours the plates were photographed. The inhibition of S. *cerevisiae* growth indicates syringomycin/syringopeptin production.

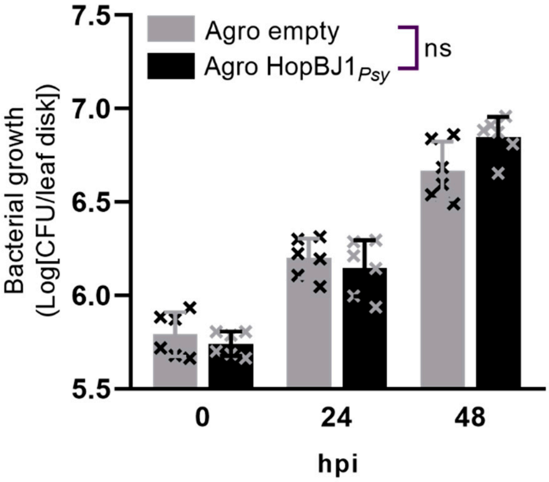

Figure S5: Evaluation of Agrobacterium growth on HopBJ1*Psy*-expressing plant tissue. To evaluate the possible effect of HopBJ1*Psy* on the plant defense, we evaluate the proliferation of Agrobacterium that carries the 35S:HopBJ1*Psy*-GFP construct (Agro HopBJ1*Psy*) or the Agrobacterium without the vector (Agro empty). Plants were agroinfiltrated as described in methods, and leaf disks were cut and ground on $MgCl_2$ 10mM at 0, 24, and 48 hours post-inoculation (hpi). The homogenized tissue was diluted and plated on LB media supplemented with Rifamplicine, Gentamicyne, and Spectinomiscine. The colony forming units (CFU) were manually counted, and the Bacterial Growth was expressed as the mean of log(CFU/leaf disk) ± SD (n=6, indicated as x in the bars). Statistical analysis does not show statistical differences between the treatments (2-way ANOVA, n.s.=no sisgnificant differences).

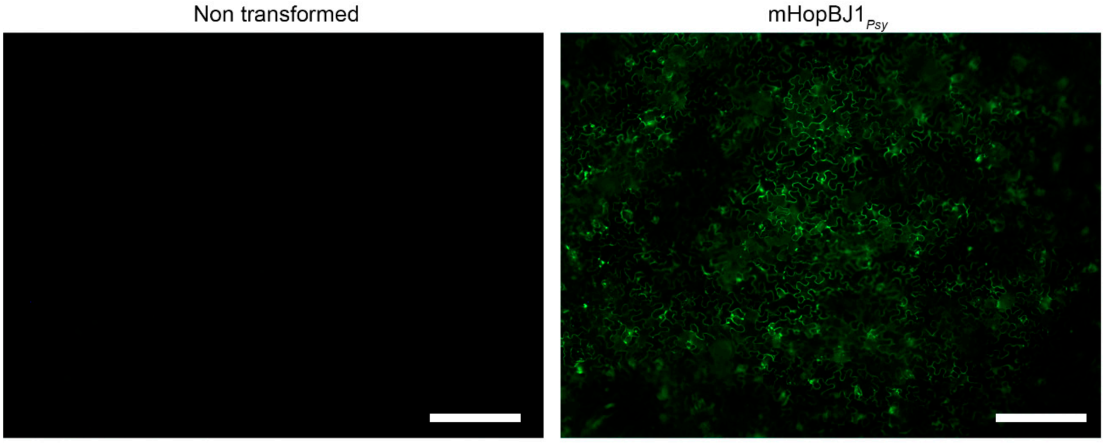

Figure S6: Evaluation of mHopBJ1*Psy* expression in tobacco plants. Tobacco plants were infiltrated with Agrobacterium carrying the mHopBJ1*Psy* gene (Cys153Ser and His171Ala, mHopBJ1*Psy*) controlled by the CaM35S promoter. As a control, untransformed Agrobacterium were infiltrated (non transformed). 24 hour post-inoculation, plants were observed on a epifluorescent microscope (Olypus IX2-UCB) coupled to a photodocumentation camera (MicroPublisher 3.3 – RTV) to detect the GFP fluorescence. Bar = 250µm.
